# Intrapartum Antibiotic Prophylaxis and Child Health Outcomes: A Systematic Review and Meta‐Analysis of Observational Studies

**DOI:** 10.1111/1471-0528.70015

**Published:** 2025-09-26

**Authors:** Maedeh Moradi, Jessica A Grieger, Xiao Tong Teong, Leonie K Heilbronn

**Affiliations:** ^1^ Adelaide Medical School University of Adelaide Adelaide South Australia Australia; ^2^ Lifelong Health Theme South Australian Health and Medical Research Institute (SAHMRI) Adelaide South Australia Australia; ^3^ Robinson Research Institute University of Adelaide Adelaide South Australia Australia

**Keywords:** autoimmune disease, child obesity, gut microbiota, intrapartum antibiotic prophylaxis

## Abstract

**Background:**

With increasing use of intrapartum antibiotic prophylaxis (IAP) for the prevention of early‐onset Group B streptococcus (GBS) infections, there is concern about its long‐term consequences on child health.

**Objectives:**

To synthesise the evidence of IAP exposure on autoimmune‐related diseases, obesity in childhood and microbial diversity in infants.

**Search Strategy:**

PubMed, Web of Science, Emcare, Embase and Scopus were searched from inception until 17 July 2025 for related observational studies.

**Selection Criteria:**

The exposure group comprised mothers with full‐term vaginal deliveries who underwent GBS screening and received IAP, while the comparator group included mothers with full‐term vaginal deliveries with GBS‐negative results and no IAP exposure.

**Data Collection and Analysis:**

Results were pooled using fixed or random‐effects meta‐analysis based on heterogeneity assessed by the *I* ^2^ statistic.

**Main Results:**

Sixteen studies were eligible to be included in the meta‐analysis. IAP exposure was associated with an increased risk of autoimmune‐related disease (6 studies, relative risks (RRs) = 1.73; 95% confidence interval [CI]: 1.08–2.78; *I* ^2^ = 94.8%). Subgroup analysis based on types of autoimmune‐related diseases showed a significant increase in the risk of atopic dermatitis (3.44; 1.60–7.37). There was a modest increase in child BMI (2 studies, standardised mean difference = 0.05; 95% CI: 0.03–0.06; *I* ^2^ = 50.09%), but not BMI *z*‐score (3 studies, 0.13; 0.03–0.29; *I* ^2^ = 72.05%) or microbiome diversity in infants (6 studies, −0.09; −0.20 to 0.02; *I* ^2^ = 0.00%) born to pregnant women exposed to IAP compared to non‐exposed women.

**Conclusion:**

IAP exposure is associated with an increased risk of autoimmune‐related disease and a modest increase in child BMI.

**Trail Registration:**

PROSPERO (CRD42023493413).

## 1 Introduction

Group B streptococcus (GBS) colonisation occurs asymptomatically in 11%–35% of women tested during pregnancy worldwide [1]. Without treatment, 11%–57% of GBS carriers will transmit the bacteria to their newborns during the intra‐partum period [2]. Among newborns, 1%–2% will develop an early‐onset GBS infection [2], although nearly half of infections occur among those born from mothers who tested negative for GBS [1]. Early‐onset GBS infection is the leading cause of morbidity and mortality in infants worldwide [3]. The global incidence is 0.41 cases/1000 live births [3, 4], accompanied by a case fatality of 5% in developed countries to 27% in developing nations [4].

There is no international consensus on the optimal strategy for identifying women eligible for intrapartum antibiotic prophylaxis (IAP) during pregnancy. One approach, recommended by The American College of Obstetricians and Gynaecologists, is a universal screening‐based approach where everyone is screened for GBS colonisation at 36–37 weeks of pregnancy, with IAP administered to all women who test positive for GBS [5, 6]. Some countries do not implement universal screening due to concerns about its cost‐effectiveness, the potential to promote microbial resistance and ineffectiveness in the prevention of late‐onset GBS infection in babies [1]. The alternative is a risk‐based approach whereby only mothers with known preceding risk factors such as preterm labour at < 37 weeks gestation, membrane rupture duration ≥ 18 h prior to birth, intrapartum temperature of ≥ 38.0°C within 24 h of giving birth, or mothers with a history of a previous newborn with GBS infection are screened and subsequently administered IAP during labour [7, 8]. Since more than 90% of women who test positive for GBS receive IAP during pregnancy, universal screening leads to widespread exposure of infants to antibiotics during the intra‐partum period [9, 10].

Antibiotic exposure during the intra‐partum period may delay the maturation of the microbiome and lead to a microbiome with lower diversity [11, 12, 13]. In turn, this may delay development of immune cell populations [14]. A study in mice showed that antibiotic treatment during pregnancy caused gut microbial dysbiosis in offspring [15]. In early life, these offspring displayed immune dysregulation, including intestinal inflammation and gut barrier disruption, leading to bacterial toxins such as lipopolysaccharide entering the bloodstream. This progression resulted in a significant increase in pro‐inflammatory Th17 cells in the blood and lungs, as well as RORγt Tregs in the lungs, which subsequently raised the offspring's risk of developing asthma [15]. IAP has been linked to increased risk of childhood autoimmune‐related diseases including atopic dermatitis, asthma and allergic rhinitis and type 1 diabetes [16, 17, 18], although results across studies have been inconsistent [16, 17, 18, 19, 20]. There is also some evidence that IAP and gut dysbiosis may increase the risk for obesity in childhood [18, 21]. The mechanisms are less well elucidated but germ‐free mice who were colonised with microbiota from donors with obesity exhibited a significantly greater increase in total body fat as compared to those colonised with microbiota from lean donors [22]. Given these emerging insights into the potential role of IAP and childhood outcomes, the aim of this systematic review and meta‐analysis is to synthesise and appraise the available evidence on the relationship between IAP exposure and childhood autoimmune‐related diseases and obesity, as well as its association with gut microbiome biodiversity and relative abundance in infants.

## 2 Methods

The Cochrane Handbook for Systematic Reviews was followed to conduct this systematic review and meta‐analysis [23]. Prior to review commencement, the study protocol was registered in PROSPERO (CRD42023493413), and is reported in accordance with the Preferred Reporting Items for Systematic Review and Meta‐Analyses (PRISMA) guidelines [24]. In conducting this meta‐analysis, we adhered to the Meta‐analysis of Observational Studies in Epidemiology (MOOSE) guidelines to ensure transparent and comprehensive reporting [25].

### 2.1 Eligibility Criteria

The following inclusion criteria were defined according to the population, exposure, comparison, outcome (PECO) framework: the population included infants or children whose mothers were exposed to IAP; the exposure was mothers who had full‐term babies via vaginal delivery, underwent GBS screening and received IAP; the comparator was mothers who had full‐term babies via vaginal delivery with negative GBS and no IAP exposure; outcomes of interest included childhood autoimmune‐related diseases, childhood obesity, infants' gut microbiota biodiversity and bacterial relative abundance. The exclusion criteria were studies that did not report caesarean section and vaginal delivery outcomes separately; studies involving mothers who received IAP for reasons other than GBS positive status, such as premature rupture of membranes; and studies that focused on antibiotic use in pregnancy rather than IAP during pregnancy.

### 2.2 Information Sources and Search Strategy

All related studies investigating the effect of IAP on autoimmune‐related diseases and obesity in childhood and gut microbiome biodiversity in newborns were searched and obtained using PubMed, Embase, Emcare, Web of Science and Scopus from inception to 30 January 2024 and updated on 17 July 2025. Briefly, search terms included key words and MeSH terms: (intrapartum antibiotic prophylaxis) AND (gastrointestinal microbiome or paediatric obesity or autoimmune‐related diseases). Google Scholar and references from recent review articles were also checked for further related papers. Published and unpublished data, including abstracts, were also included to minimise publication bias and provide a more comprehensive picture of the evidence. Detailed search terms for each database are reported in Table S1. Searches were limited to human studies, and no restriction was applied on the language.

### 2.3 Study Selection

**FIGURE 1:**
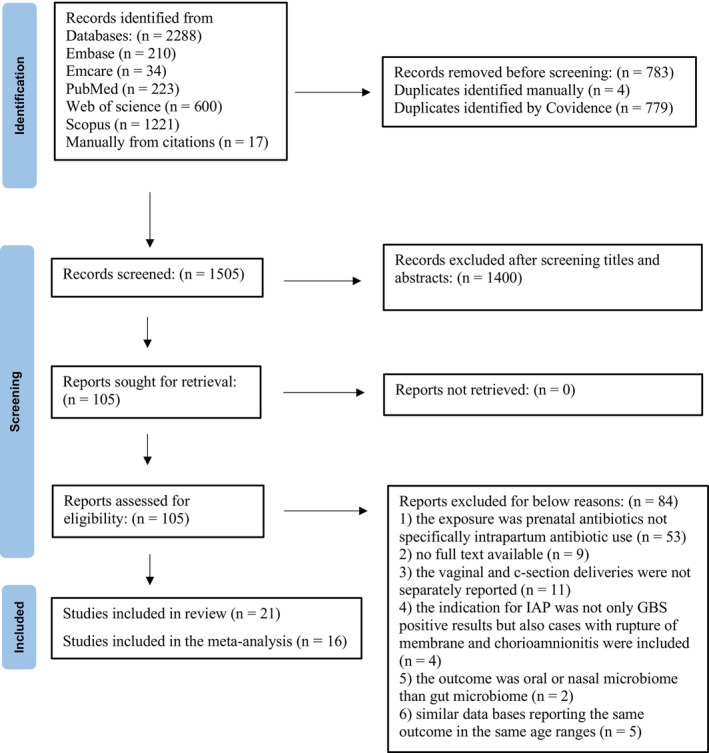
The PRISMA flow chart.

The PRISMA flow chart is shown in Figure 1. All records retrieved through electronic searching were imported into Covidence, systematic review software (Veritas Health Innovation, Melbourne, Australia; www.covidence.org) for title and abstract screening. Two independent reviewers (MM, XT) screened all titles and abstracts according to predefined inclusion and exclusion criteria. If the record was unclear from the title and abstract alone, the record was included for full‐text screening, with reasons for exclusion documented (Figure 1). The same reviewers independently undertook full‐text screening, and any ambiguities were resolved through discussion or with a third reviewer (JG).

### 2.4 Data Extraction

Data from eligible articles was extracted by MM onto a Microsoft Excel spreadsheet that included author, publication year, country, study design, number of participants, pre‐pregnancy body mass index (BMI), child's age at follow‐up, outcome assessment, confounding variables, risk estimates with 95% confidence intervals (CIs) or mean (SD). If several risk estimates were provided, those in the full multivariable adjusted model are considered. When data were presented in graphical form rather than as numerical values, data was extracted by MM using WebPlotDigitizer software and XT checked the data extracted for accuracy. Two corresponding authors from two papers were contacted twice to obtain additional information about outcomes of child obesity.

### 2.5 Assessment of Risk Bias

The Newcastle–Ottawa quality assessment scale was used to estimate the risk of bias in cohort studies [26]. A maximum of 9 points was given based on 8 items in 3 scoring domains, including the selection of the study groups (4 points), comparability of the groups (2 points) and assessment of outcome (3 points). The maximum points that could be awarded to each study was 9, and a score ≥ 7 was considered high quality [27].

### 2.6 Grading of Recommendations, Assessment, Development and Evaluation

The certainty of the evidence was evaluated using the GRADE tool [28], which considers five key domains: risk of bias in individual studies, inconsistency, indirectness, imprecision and publication bias. Each study was rated by MM as having a high, moderate, low or very low level of certainty based on these evaluative criteria.

### 2.7 Data Synthesis and Analysis

The meta‐analysis was performed by pooling the multivariable‐adjusted relative risks (RRs), hazard risks (HRs) or odds ratios (ORs) of IAP exposed group compared with the non‐exposed group and the corresponding 95% CIs. When there were separate findings for gut microbiome biodiversity in age groups < 3 or ≥ 3 months, data were extracted for both results and were included separately in the meta‐analysis [29]. When the study reported risk estimates on asthma and atopic dermatitis separately, the estimates were pooled for each subtype using a fixed‐effects model to obtain the composite outcome to be used in the meta‐analysis [18]. We used a fixed‐effects model within individual studies because asthma and atopic dermatitis are closely related conditions. In studies reporting allergic rhinitis along with other subtypes of autoimmune‐related diseases, allergic rhinitis was reported as a separate outcome [19]. In cases where the study did not report RR, we calculated it by dividing the probability of the event occurring in the exposed group by the probability of the event occurring in the control group [30]. The reported HRs were considered equal to RRs [31]. For measures of BMI, BMI *z*‐score and gut microbiome biodiversity, the mean difference and standard deviation between the intervention and control group were used. If the standard deviation was not reported, values from the corresponding 95% CIs or interquartile range were used according to Cochrane handbook [32, 33]. When the study reported an effect size for BMI and BMI *z*‐scores, both measures were included in their respective meta‐analysis [18]. One of the two authors responded to the inquiries regarding the measures of BMI and BMI *z*‐score [18]. Heterogeneity was assessed using the Cochrane test and the *I* ^2^ statistic, with an *I* ^2^ value > 50% considered indicative of potentially significant statistical heterogeneity [34]. To consider between‐study heterogeneity, a random‐effects model (DerSimonian–Laird) to calculate the overall effect size was conducted. Sensitivity analyses were conducted to evaluate the effect of removing any single study from the meta‐analysis. Sensitivity analysis was performed excluding studies that did not adjust for confounding factors to reveal whether such studies have an impact on the summary effect [16, 35]. To investigate the potential sources of heterogeneity, subgroup analysis was performed to evaluate the robustness of results according to age of newborns (< 3 or ≥ 3 months), age of child (< 3 or ≥ 3 years), receiving single or mixed antibiotics as IAP, and subtypes of autoimmune‐related diseases (composite outcomes or atopic dermatitis or allergic rhinitis). Publication bias was not assessed as all analyses were based on < 10 studies [23]. All statistical analyses were performed with Stata, version 18 (Stata Corp). *p* values of < 0.05 were considered significant.

## 3 Results

A total of 2288 potentially relevant papers were identified from the literature search, of which 783 duplicates were removed and 105 papers underwent full‐text review. During full‐text review, 84 papers were excluded, 21 papers were included in the systematic review and 16 papers (20 effect sizes) in the meta‐analysis (Figure 1).

### 3.1 Characteristics of Included Studies

Characteristics of the 21 included studies are shown in Tables 1, 2 and S2. The sample size ranged from 13 to 117 772 women–child dyads. Included studies reported on 26 467 participants in the IAP exposed group and 110 120 participants in the non‐exposed group. Follow‐up periods ranged from 6 days up until 10 years. Eleven studies were from North America, 7 from Europe and 2 from Asia. Twelve studies adjusted for at least one confounder, and nine studies did not make any adjustments. Types of antibiotics used in the IAP exposed group included single antibiotics or combinations of antibiotics.

**TABLE 1:** Basic characteristics of eligible studies for autoimmune‐related diseases.

First author (year)	Design child age at follow‐ up (y)	Mothers exposed to IAP/non‐exposed	Tye of IAP	Outcome assessment method	Adjusted variables	Effect measure type	Childhood auto‐immune diseases relative effect (95% CI)
Ainonen et al. (2024) US	Retrospective cohort 1 year		Penicillin G		Adjusted for different maternal and neonatal factors	HR (95% CI)	Composite outcome 1.08 (0.96, 1.20) Allergic rhinitis 1.12 (0.83, 1.50) Increased risk of type 1 diabetes, rheumatoid diseases and autoimmune eye diseases
Hutton et al. (2023) Canada	Prospective cohort 1 year	25/185	Penicillin G, ampicillin, clindamycin, ceftriaxone, gentamicin	Physician‐diagnosed	Breastfeeding	RR	Atopic dermatitis 2.80 (1.14, 6.85)
Zhang et al. (2023) China	Retrospective cohort 3 years	256/243	Penicillin G or ampicillin or cefazolin or clindamycin	Parental report	Parity	RR	Allergic rhinitis 2.36 (1.32, 4.21)
Hong et al. (2022) China	Retrospective cohort 2 years	164/1346	Penicillin G or ampicillin or cefazolin	Diagnosed according to Williams standards	Breastfeeding, neonatal antibiotic exposure, parity	RR using N of events	Atopic dermatitis 6.56 (3.27, 5.14)
Dhudasia et al. (2021) US	Retrospective cohort 5 years	1919/6989		Defined as prescribed diagnosis codes	Breastfeeding, neonatal antibiotic exposure, parity	RR using N of events	Allergic rhinitis 0.85 (0.62, 1.18) Composite outcome 1.21 (0.96, 1.52)
Wohl et al. (2015) US	Retrospective cohort 2 years	128/364	Penicillins, cephalosporins, macrolides and aminoglycosides	Medical records or	None	RR	Atopic dermatitis 1.99 (1.13, 3.49)

**TABLE 2:** Basic characteristics of eligible studies for childhood obesity outcome.

First author (year)	Design	Child age at follow‐up	Population mothers exposed to IAP/non‐exposed	Tye of IAP	Outcome assessment method	Adjusted variables	Childhood obesity mean change (95% CI)
Hutton et al. (2023) Canada	Prospective cohort	1 years	40/172	Penicillin G, ampicillin, clindamycin, ceftriaxone and/or gentamicin	Medical records	Breastfeeding	No change in BMI *z*‐score No change in BMI
Sidell et al. (2023) US	Retrospective cohort	10 years	22 528/95 244	Penicillin G, ampicillin, cefazolin, clindamycin and/or vancomycin	Medical records	Other prenatal antibiotic exposure, breastfeeding, neonatal antibiotic exposure, maternal disease	BMI increased by 0.14 unit
Klancic et al. (2022) Canada	Prospective cohort	1 years	432/830	NR	Medical records	Maternal disease	BMI *z*‐score increased by 0.25 unit
Metz et al. (2020) US	Retrospective cohort	2–5 years	786/3916	Penicillin G, ampicillin, cefazolin, clindamycin and/or vancomycin	Medical records	Maternal BMI, child weight at birth, black race and gestational age at delivery	No change in BMI *z*‐score

### 3.2 Results of the Meta‐Analyses

In the meta‐analysis, six studies with eight effect sizes evaluated the effects of IAP on the risk of autoimmune‐related disease, four studies with five effect sizes examined child obesity and six studies with seven effect sizes examined gut microbiota alpha biodiversity.

#### 3.2.1 IAP and Child Autoimmune‐Related Diseases

**FIGURE 2:**
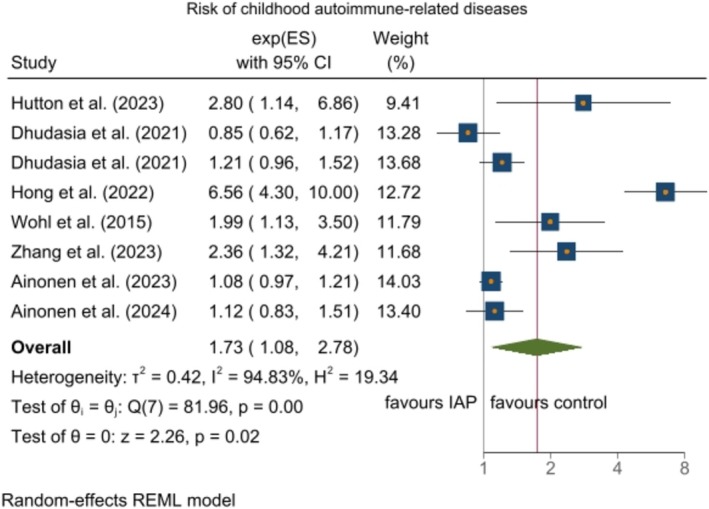
The effect of IAP on risk of childhood autoimmune‐related diseases.

Six cohort studies were included that investigated the association between IAP and autoimmune‐related disease in children. Due to the substantial heterogeneity observed among the included studies (*I* ^2^ = 94.8%; *p* < 0.0001), a generic inverse‐variance random‐effects model was employed for determining the pooled RRs. There was evidence of an increased risk of autoimmune‐related disease in the IAP exposed group as compared to the control group (RR = 1.73; 95% CI: 1.08–2.78, *p* = 0.006) (Figure 2). In subgroup analysis comparing children aged < 3 versus ≥ 3 years, the pooled RR of autoimmune‐related diseases was 3.42 (95% CI: 1.48–7.91; *I* ^2^ = 83.01%; *p* = 0.003) with high heterogeneity for age < 3 years and age ≥ 3 years (RR = 1.14; 95% CI: 0.95–1.37; *I* ^2^ = 59.6%; *p* = 0.042) (Figure S2a). In subgroup analysis comparing RR based on types of autoimmune‐related diseases, only the risk of atopic dermatitis was significant (RR = 3.44; 95% CI: 1.60–7.37) while heterogeneity remained high (*I* ^2^ = 79.4%; *p* = 0.003). However, in the case of other types of autoimmune‐related disease including composite outcomes and allergic rhinitis, no significant association was found. Heterogeneity remained high in the atopic dermatitis and allergic rhinitis subgroups and dropped to zero in the composite outcome subgroup (Figure S2b). Excluding any single study arm from the meta‐analysis did not alter the pooled estimate. Results from sensitivity analysis showed that no single study biased the overall estimates regarding the effect of IAP. Sensitivity analysis showed that excluding the study by Wohl et al. which did not adjust for any confounders, did not materially change the results. Furthermore, excluding the study by Ainonen et al. which reported the association using HR rather than RR, also had no significant impact on the overall results, indicating the robustness of our findings (Figure S2c).

#### 3.2.2 IAP and Child Obesity

**FIGURE 3:**
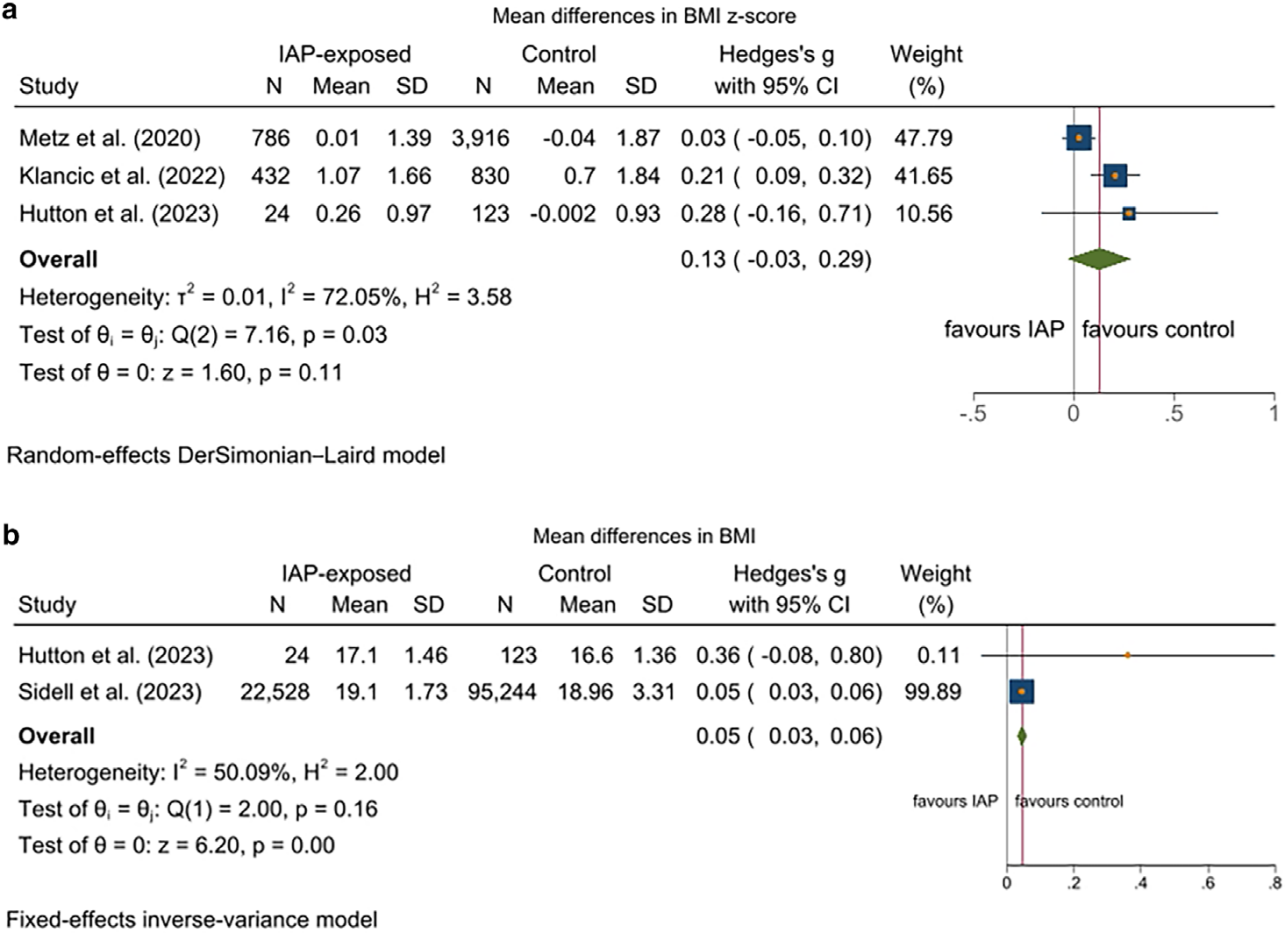
(a) The effect of IAP on childhood obesity measured by BMI *z*‐score. (b) The effect of IAP on childhood obesity measured by BMI.

Four cohort studies (one study reporting both BMI and BMI *z*‐score) were included in the meta‐analysis. Comparing the IAP‐exposed group with the control group, (BMI (SMD) = 0.05; 95% CI: 0.03–0.06; *p* < 0.0001) was higher in children whose mothers were among the IAP‐exposed group compared to the control group, while BMI *z*‐score did not change in IAP‐exposed compared to the control group (SMD = 0.13; 95% CI: −0.03 to 0.29; *p* = 0.11) (Figure 3a,b). Moderate and high heterogeneity was observed among the included studies reporting results on BMI (*I* ^2^ = 50.0%; *p* = 0.16) and BMI *z*‐score (*I* ^2^ = 72.07%; *p* = 0.03), respectively. Thus, a random‐effects model was used to calculate the SMD for BMI *z*‐score, and a fixed model was used for BMI.

#### 3.2.3 IAP And Infant Gut Microbiome Biodiversity

Six cohort studies were included in the meta‐analysis. There was no significant heterogeneity among studies assessing gut microbiota biodiversity in infants; hence, an inverse‐variance standard‐effect model was used to determine the pooled results. Results showed no statistically significant difference between IAP exposed and control groups (SMD = −0.09; 95% CI: −0.20 to 0.02, *I* ^2^ = 0.00%; *p* = 0.12) (Figure S1a). In the subgroup analysis by type of IAP and infant's age, the pooled effect sizes indicated no significant differences in gut microbiome biodiversity across all subgroups. The heterogeneity remained low across these subgroups, indicating that the type of IAP and infant's age are not sources of heterogeneity in this context (Figure S1b,c). Furthermore, following sensitivity analysis, systematic removal of each cohort study from the analysis did not change the results (Figure S1d). The studies evaluating the effects of IAP on gut microbiota relative abundance demonstrated that the majority reported reduced abundance of *Bifidobacterium* within the Actinobacteria phylum (6 of 8 studies), reduced Bacteroidetes phylum (6 of 8 studies), increased abundance of different genera of bacteria within the phylum Firmicutes (5 of 8 studies) and increased abundance of *Escherichia coli* or Enterobacteriaceae within the Proteobacteria phylum (3 of 4 studies) (Figure S1e).

### 3.3 Risk of Bias of Included Studies

Total NOS scores ranged from 5 to 9. The NOS rating indicated that 95% of the studies were of high quality (score ≥ 7), with 1 study scoring a 5 (Table S3). Except for one, all studies received a rating of 4 for selection, indicating a high level of representativeness of the selected population and accuracy in identifying the exposure of interest. There was significant variability in the criteria for comparability, with 15 studies receiving a score of 0 on this 2‐point scale. All included studies received a score of 3 in the outcomes assessment criterion.

### 3.4 Certainty of Evidence

The GRADE protocol was applied to assess the certainty of evidence. Regarding IAP exposure and its association with childhood autoimmune‐related diseases, BMI *z*‐score and infant gut microbiota, the quality of evidence was determined to be of very low certainty due to very serious limitations in inconsistency and serious limitations in indirectness and imprecision. The quality of evidence for the association between IAP exposure and child BMI was rated as low (Table S4).

## 4 Discussion

### 4.1 Main Findings

The current meta‐analysis utilising 11 619 mother‐infant dyads indicates that IAP exposure is associated with a 1.70‐fold increased risk of childhood autoimmune‐related diseases. The quantitative synthesis assessing IAP exposure on child obesity showed a modest increase in BMI, while BMI *z*‐score was not statistically different. The evidence for child obesity was limited to only four studies, which had a very low certainty. We did not find any clear differences in the alpha biodiversity of the infant gut microbiome between the IAP‐exposed and non‐exposed groups.

### 4.2 Strengths and Limitations

The strengths of the study are the inclusion of only uncomplicated pregnancies to mitigate the effects of confounding factors related to infection, preeclampsia, gestational diabetes or other inflammatory conditions [36]. The meta‐analysis only included vaginal delivery as caesarean section has a known impact on gut microbiota [37, 38, 39, 40, 41, 42, 43] and studies where women had at least 4 h of IAP exposure. Preterm infants or infants born to mothers with PROM were also excluded as there is evidence that neonates with a maternal history of PROM are at high risk of developing neonatal sepsis [44, 45, 46]. We also excluded four studies where multiple publications were found by the same research group to avoid potential participant overlap [47, 48, 49, 50].

There are several limitations with the individual studies and thus findings should be interpreted with caution. While the primary aim of this study was to assess the effects of IAP on autoimmune‐related diseases, most of the eligible studies focused on allergic rhinitis, atopic dermatitis or composite outcomes. Hence, the number of studies reporting on other autoimmune diseases, such as type 1 diabetes, was not sufficient to conduct a meta‐analysis. Not all included studies have accounted for potential confounders, including antibiotic treatment during pregnancy, infant exposure to antibiotics, pet exposure, maternal history of allergic disease, parity, maternal age, maternal BMI, education and breastfeeding [51, 52, 53]. This inconsistency may have introduced high heterogeneity, potentially affecting the results. The lack of differentiation between the different classes of antibiotics used could introduce bias into the results, as each class has been shown to affect outcomes in distinct ways. For example, one study demonstrated that among IAP antibiotics, penicillins have a sustained impact on the microbiome throughout the first year of life, whereas the effects of multi‐class antibiotics typically do not persist beyond 6 weeks [29]. Another limitation of the current study is that measures reported by one study were reported as HR [54]. This is an approximation, and its validity decreases as event rates increase or follow‐up time lengthens [31]. These factors, coupled with the retrospective design of most studies, small sample sizes in several studies and the use of different diagnostic methods, are additional limitations of the studies included in the current meta‐analysis.

### 4.3 Interpretation of Findings

The results of this meta‐analysis on childhood autoimmune‐related diseases align with findings from meta‐analyses reporting associations between prenatal antibiotic exposure and childhood autoimmune‐related diseases [55, 56, 57]. However, compared to those meta‐analyses investigating IAP solely during vaginal delivery, as in our meta‐analysis, reduces confounding factors, including other maternal infections or complications, are reduced, which could independently influence neonatal immune outcomes. A prospective cohort study of 49 299 women reported an association between IAP and other childhood autoimmune‐related diseases, including type 1 diabetes, rheumatoid diseases and autoimmune eye conditions [54]. While these outcomes were not included in the current meta‐analysis due to a lack of prior studies to pool them together, the results on asthma, atopic dermatitis and allergic rhinitis were considered in our meta‐analysis, and other autoimmune outcomes were reported in Table 1. The underlying mechanism that links IAP to autoimmune‐related disease is rooted in its potential to disrupt the early development of the infant's gut microbiome. In the current study, although we did not observe any significant effect of IAP during vaginal delivery on gut microbiome biodiversity, results from the systematic review indicate that most studies reported significant changes in gut microbiome composition (Figure S1e). IAP is proposed to increase the risk of autoimmune‐related diseases in children by altering the microbial colonisation process, which is critical for the maturation of the immune system. This disruption delays the progression of the immune response from a T helper 2‐dominant state, characteristic of early infancy, to a more balanced T helper 1‐dominant response [58]. The prolonged T helper 2 bias prevents the immune system from achieving a balanced state, thereby increasing the likelihood of immune dysregulation and the development of autoimmune‐related diseases [59, 60, 61]. Furthermore, gut microbiome dysbiosis can increase the risk of autoimmune diseases by compromising gut barrier integrity. Dysbiosis disrupts the protein complexes in intestinal tight junctions, such as occludins and claudins, leading to increased intestinal permeability [62]. This allows microbial products, such as bacterial lipopolysaccharides and flagellin, to translocate into the bloodstream, triggering systemic inflammation. The resulting systemic inflammation contributes to immune dysregulation, which can promote the development of autoimmune‐related diseases [62].

The current meta‐analysis on autoimmune outcomes is limited by the high heterogeneity of included studies. To partly address this, we conducted a subgroup analysis based on children's age [63]. By age 3 years, children generally have more mature immune systems and gut microbiota resembling adult‐like patterns. This distinction helps us explore whether early immune programming effects persist or diminish after this stage of life [64]. The subgroup analysis indicated that IAP increased the risk of autoimmune‐related diseases in children < 3 years but was not observed in older children. This finding contrasts with meta‐analyses on prenatal antibiotic exposure, which have reported a higher risk of allergic diseases in older children compared to younger children [65, 66]. In one study included in the older children subgroup of this meta‐analysis, children diagnosed with asthma during their first year of life were excluded to reduce the likelihood of including early wheezing caused by viral infections [19]. This exclusion may have led to an underestimation of asthma risk in this subgroup, as cases that could have been diagnosed as asthma were excluded. While previous studies reported inconsistent findings regarding the effects of breastfeeding on autoimmune‐related diseases [20, 66, 67], only three studies in this meta‐analysis accounted for breastfeeding as a confounding variable [17, 18, 19]. However, the sensitivity analysis showed that including breastfeeding as a confounding factor did not alter the overall results, suggesting that the association observed in this meta‐analysis is robust and independent of breastfeeding practices.

Our results regarding the effect of IAP exposure on BMI in childhood align with one recent meta‐analysis evaluating the effect of prenatal antibiotic exposure [68], but not an umbrella review synthesising evidence from five meta‐analyses [69]. As already highlighted, underlying maternal conditions could have influenced their results, and the certainty of evidence was rated as very low [69]. In the current meta‐analysis, the effect size with BMI was modest, and the quality of the included studies was also low. We did not detect a relationship between IAP exposure and BMI *z*‐score in contrast to a recent study that reported an association between IAP exposure, but not prenatal antibiotic use and BMI *z*‐score [70]. Studies in humans are needed to determine if there is a relationship between IAP exposure and risk for childhood obesity.

We could not detect an effect of IAP on infant microbiome biodiversity as assessed by Shannon alpha diversity. These findings align with a previous meta‐analysis which included women with ruptured membranes or who were GBS‐positive [51]. The Shannon index provides insight into overall microbial diversity but does not reflect taxon‐specific imbalances [71]. This could mask the relationship between the effects of IAP exposure and certain microbes which may have been impacted [72]. We could not conduct a meta‐analysis on the abundance of the intestinal microbiota at the phylum level given the limitations of existing literature. A previous systematic review found reduced abundance of Bifidobacterium within the Actinobacteria phylum, reduced Bacteroidetes phylum, increased abundance of different genera of bacteria within the phylum Firmicutes, and increased abundance of *E. coli* or Enterobacteriaceae within the Proteobacteria phylum reported from 426 infants exposed to IAP [73]. These results are consistent with the current systematic review in which we included only vaginally delivered newborns, since differences in gut microbiome diversity exist between newborns delivered by caesarean sections and those delivered vaginally [38]. Several studies have suggested that early‐life exposures have a more pronounced effect on the gut microbiota up to 3 months of age [37, 74]. However, in the present study, there was no difference in the meta‐analysis results between the age subgroups (< 3 months vs. ≥ 3 months). The spectrum of action of antibiotics used may also differentially impact infant gut microbiome composition and biodiversity. However, subgroup analysis by IAP subtype, such as single‐agent penicillin or broad‐spectrum, did not impact study results. Broad‐spectrum antibiotics have been shown previously to have a stronger effect on atopic and metabolic disorders in children as compared to narrow‐spectrum antibiotics [75]. Large‐scale trials are needed to clarify the link between IAP exposure and gut microbiome in infancy and on longer‐term follow‐up.

## 5 Conclusion

The present meta‐analysis suggests that IAP as prophylactic practice is not without any health consequences to infants and children. Children born to mothers exposed to IAP showed a significantly higher risk of developing autoimmune‐related diseases compared to those who were not exposed. A quantitative analysis of IAP exposure and childhood obesity indicated a slight increase in BMI among children born to exposed mothers. Additionally, no significant differences were observed in the gut microbiome's alpha diversity between IAP‐exposed and unexposed infants. The present meta‐analysis suggests there are potential health consequences of IAP on the long‐term health of infants and children. It is crucial for future research to focus on understanding these enduring effects more comprehensively by conducting prospective and follow‐up data collection and exploring underlying mechanisms to better understand these effects, and weigh that against the potential risks.

## Author Contributions

**Maedeh Moradi:** software, data curation, writing – original draft preparation, visualisation, formal analysis, investigation. **Jessica A Grieger:** methodology, supervision, validation, writing – reviewing and editing. **Xiao Tong Teong:** data curation, writing – reviewing and editing. **Leonie K Heilbronn:** conceptualisation, supervision, writing – reviewing and editing.

## Ethics Statement

The authors have nothing to report.

## Conflicts of Interest

The authors declare no conflicts of interest.

## Supporting information


**Figure S1a:** Mean differences in gut microbiome biodiversity reported as Shannon index.

**Figure S1b:** Subgroup analysis IAP and mean differences in gut microbiome diversity in infants aged ≤ 3 months versus > 3 months.

**Figure S1c:** Subgroup analysis of mean differences in gut microbiome diversity by narrow‐spectrum IAP versus broad‐spectrum IAP exposure.

**Figure S1d:** Sensitivity analysis of gut microbiome biodiversity reported as Shannon index.

**Figure S1e:** Effects of IAP on the relative abundance of the gut microbiota.

**Figure S2a:** Subgroup analysis of IAP and risk of childhood autoimmune‐related disease in children aged < 3 years versus > 3 years.

**Figure S2b:** Subgroup analysis of IAP and risk of childhood autoimmune‐related disease by type of outcome.

**Figure S2c:** Sensitivity analysis of IAP and risk of childhood autoimmune‐related disease.

**Table S1:** Search terms and strategies for literature identification in databases.

**Table S2:** Basic characteristics of eligible studies for infants' gut microbiome outcomes.

**Table S3:** Newcastle‐Ottawa quality assessment scale for included studies.

**Table S4:** GRADE profile of IAP and child autoimmune‐related disease, child BMI and BMI *z*‐score and infant gut microbiome.

## Data Availability

The data that support the findings of this study are available on request from the corresponding author. The data are not publicly available due to privacy or ethical restrictions.
