# Supplementary figures and images for: Intrapartum Antibiotic Prophylaxis and Child Health Outcomes: A Systematic Review and Meta‐Analysis of Observational Studies
Source: BJOG. 2025 Sep 26;133(4):556–67. doi: 10.1111/1471-0528.70015 (PMC12884238; doi:10.1111/1471-0528.70015)

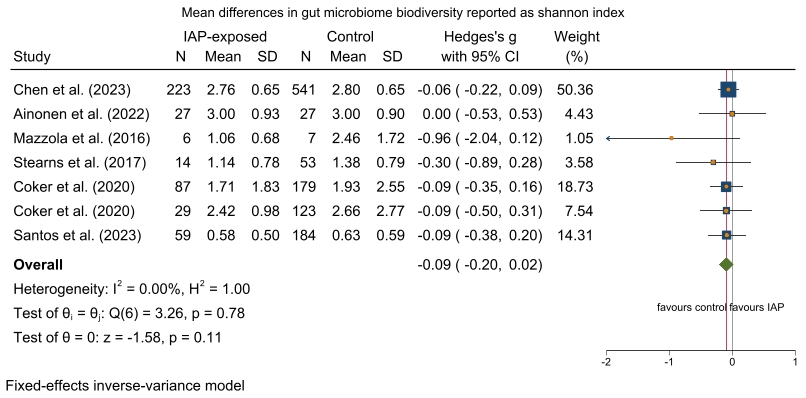

Supplement: Supplementary file 1 — Figure S1a: Mean differences in gut microbiome biodiversity reported as Shannon index. [file BJO-133-556-s005.jpg]

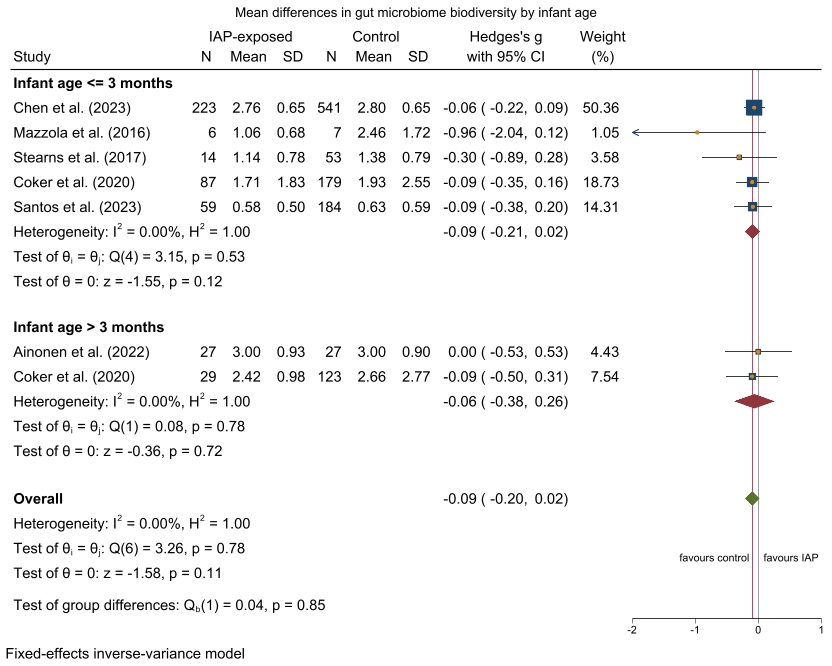

Supplement: Supplementary file 2 — Figure S1b: Subgroup analysis IAP and mean differences in gut microbiome diversity in infants aged ≤ 3 months versus > 3 months. [file BJO-133-556-s010.jpg]

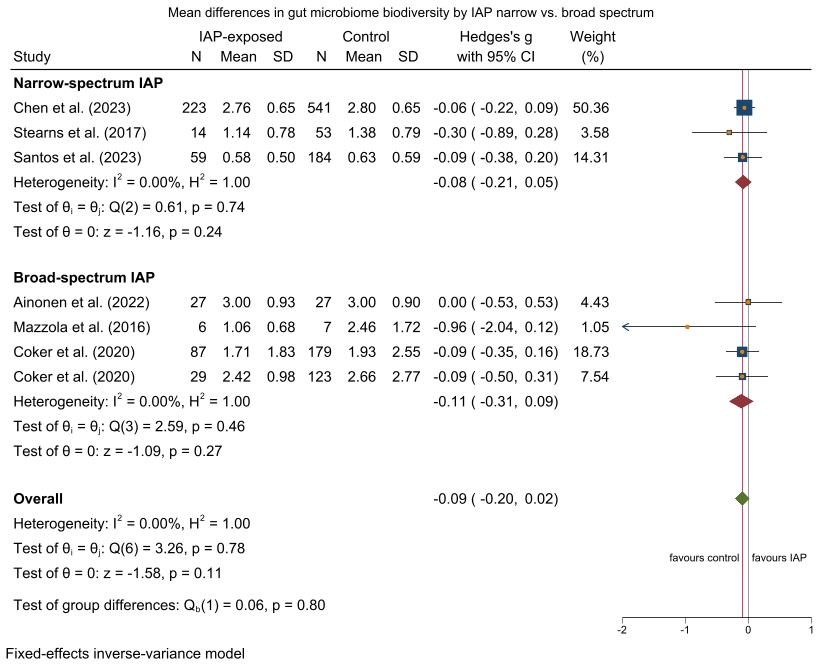

Supplement: Supplementary file 3 — Figure S1c: Subgroup analysis of mean differences in gut microbiome diversity by narrow‐spectrum IAP versus broad‐spectrum IAP exposure. [file BJO-133-556-s003.jpg]

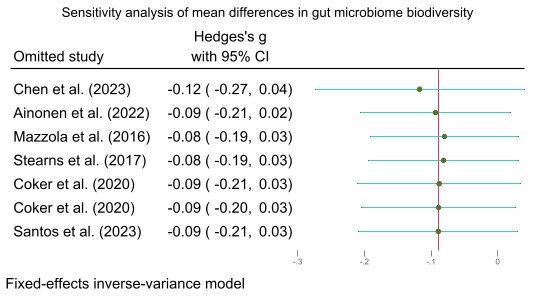

Supplement: Supplementary file 4 — Figure S1d: Sensitivity analysis of gut microbiome biodiversity reported as Shannon index. [file BJO-133-556-s007.jpg]

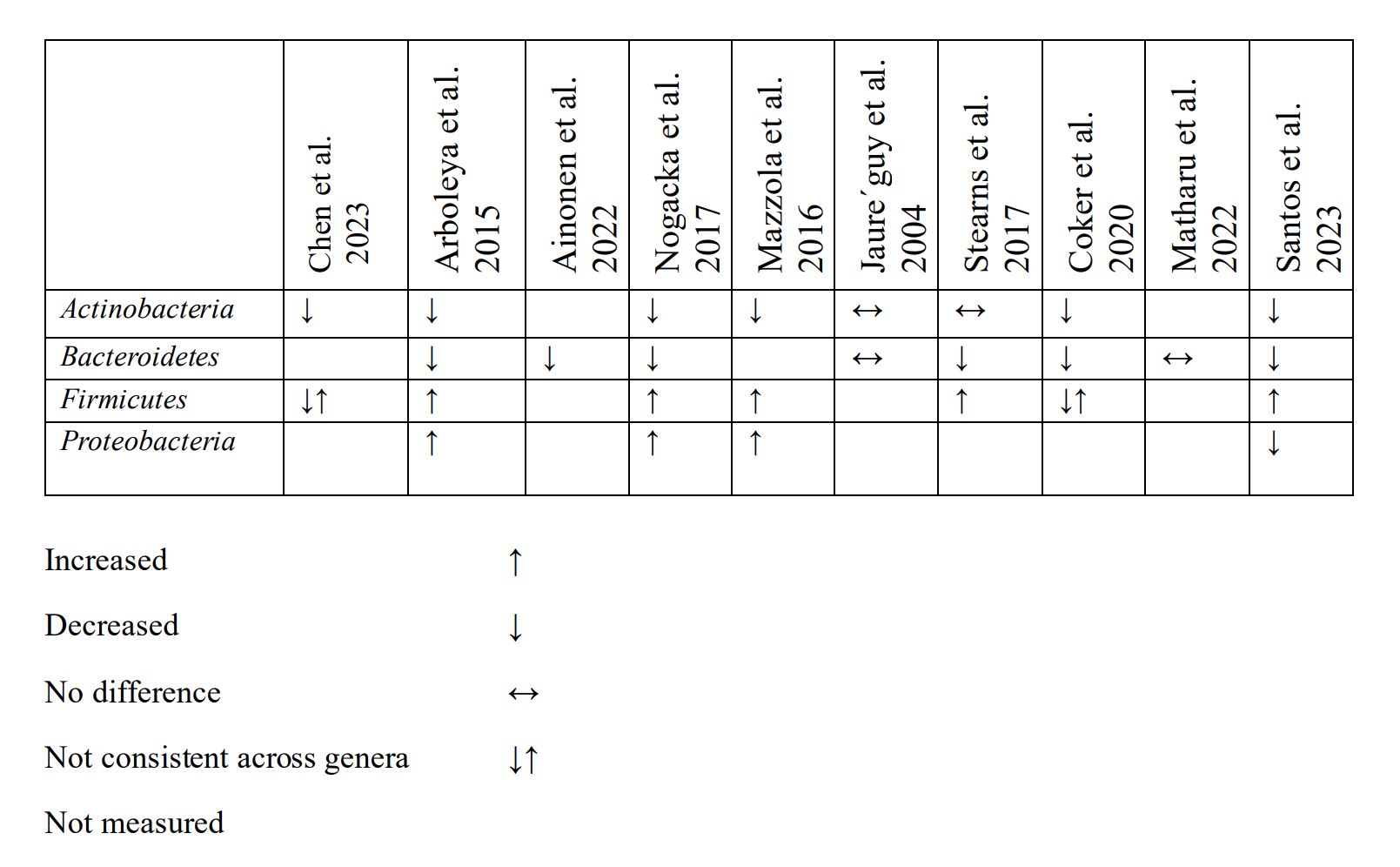

Supplement: Supplementary file 5 — Figure S1e: Effects of IAP on the relative abundance of the gut microbiota. [file BJO-133-556-s011.jpg]

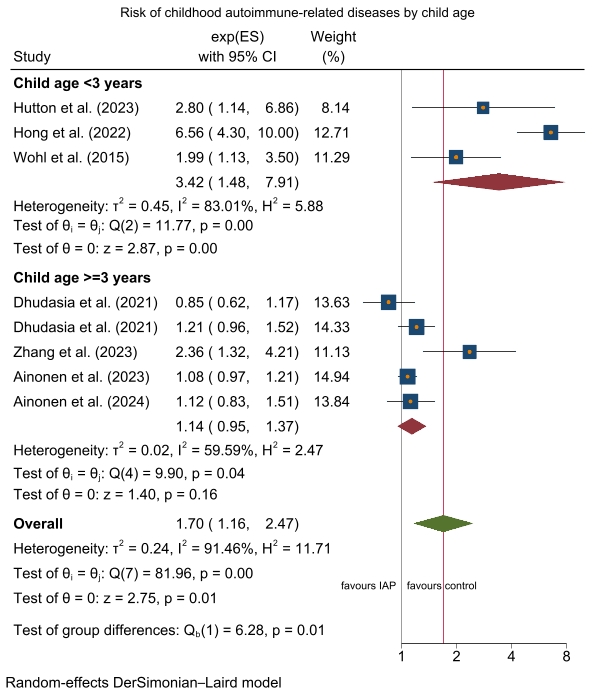

Supplement: Supplementary file 6 — Figure S2a: Subgroup analysis of IAP and risk of childhood autoimmune‐related disease in children aged < 3 years versus > 3 years. [file BJO-133-556-s009.jpg]

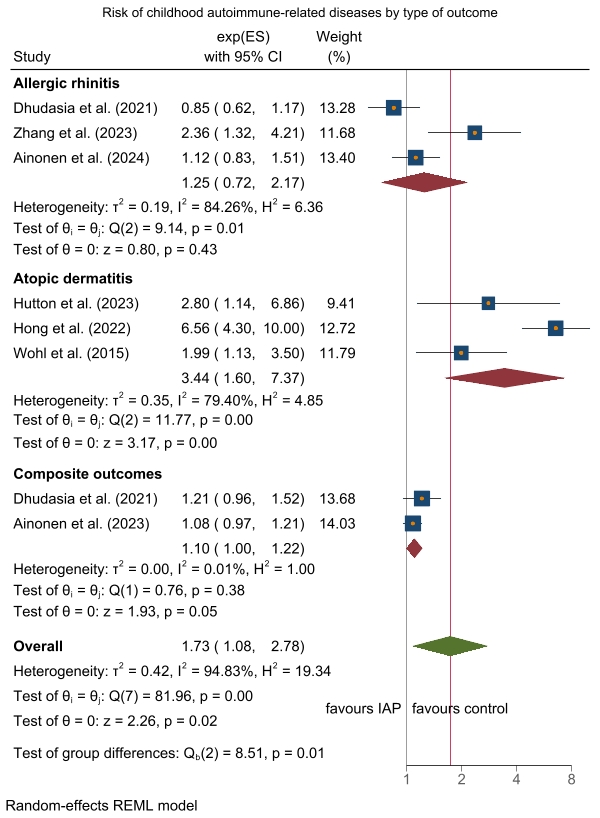

Supplement: Supplementary file 7 — Figure S2b: Subgroup analysis of IAP and risk of childhood autoimmune‐related disease by type of outcome. [file BJO-133-556-s012.jpg]

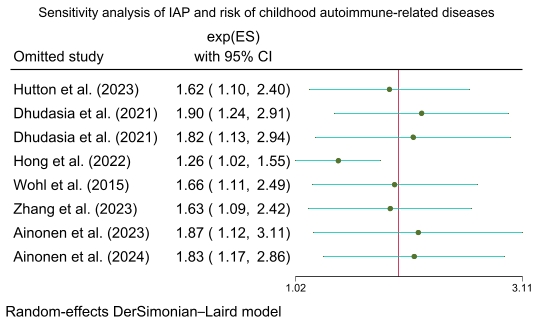

Supplement: Supplementary file 8 — Figure S2c: Sensitivity analysis of IAP and risk of childhood autoimmune‐related disease. [file BJO-133-556-s008.jpg]
