# Supplementary material for: Intrapartum Antibiotic Prophylaxis and Child Health Outcomes: A Systematic Review and Meta‐Analysis of Observational Studies
Source: BJOG. 2025 Sep 26;133(4):556–67. doi: 10.1111/1471-0528.70015 (PMC12884238; doi:10.1111/1471-0528.70015)
Supplement: Supplementary file 9 — Table S1: Search terms and strategies for literature identification in databases. [file BJO-133-556-s001.docx]

**Embase search strategy**

| 1 | ((antibiotic prophylaxis.sh OR group B streptococcal infection.sh) AND (exp labor OR exp birth OR pregnancy.sh)) OR intrapartum antibiotic prophylaxis.ti,ab OR (antibiotic* adj3 (labo$r OR prenatal OR pregnancy)).ti,ab | 3201 |
| --- | --- | --- |
| 2 | (intestine flora.sh AND (exp infant OR child.sh)) OR ((Gastrointestinal microbio* OR gut microbio* OR gut flora OR gut microflora OR gastrointestinal flora OR gastrointestinal microflora OR intestinal flora OR intestinal microflora OR gastric microbio* OR enteric bacteria) AND (infan* OR child*)).ti,ab | 13500 |
| 3 | childhood obesity.sh OR ((body mass.sh OR obesity.sh) AND exp child) OR (child* obesity OR child obesity OR childhood obesity OR p?ediatric obesity).ti,ab | 129370 |
| 4 | ((autoimmune disease.sh OR exp hypersensitivity OR food allergy.sh OR eczema.sh OR exp asthma OR exp atopic dermatitis OR exp rhinitis) AND exp child) OR ((autoimmun* OR hypersensitiv* OR asthma OR eczema OR atopic dermatitis OR food allerg* OR rhinitis) adj4 (child* OR p?ediatric)).ti,a | 161678736 |
| 5 | (exp animal OR animal experiment.sh OR nonhuman.sh) NOT ((exp animal OR animal experiment.sh OR nonhuman.sh) AND (exp human OR human experiment.sh)) | 7276759 |
| 6 | 1 AND 2 | 135 |
| 7 | 6 NOT 5 | 130 |
| 8 | 1 AND 2 AND 3 | 18 |
| 9 | 8 NOT 5 | 19 |
| 10 | 1 AND 2 AND 4 | 34 |
| 11 | 10 NOT 5 | 22 |
| 12 | 1 AND 3 | 93 |
| 13 | 12 NOT 5 | 89 |
| 14 | 7 OR 9 OR 11 OR 13 | 210 |

**Emcare search strategy**

| 1 | ((antibiotic prophylaxis.sh OR group B streptococcal infection.sh) AND (exp labor OR exp birth OR pregnancy.sh)) OR intrapartum antibiotic prophylaxis.ti,ab OR (antibiotic* adj3 (labo$r OR prenatal OR pregnancy)).ti,ab | 910 |
| --- | --- | --- |
| 2 | (intestine flora.sh AND (exp infant OR child.sh)) OR ((Gastrointestinal microbio* OR gut microbio* OR gut flora OR gut microflora OR gastrointestinal flora OR gastrointestinal microflora OR intestinal flora OR intestinal microflora OR gastric microbio* OR enteric bacteria) AND (infan* OR child*)).ti,ab | 3301 |
| 3 | childhood obesity.sh OR ((body mass.sh OR obesity.sh) AND exp child) OR (child* obesity OR child obesity OR childhood obesity OR p?ediatric obesity).ti,ab | 37768 |
| 4 | ((autoimmune disease.sh OR exp hypersensitivity OR food allergy.sh OR eczema.sh OR exp asthma OR exp atopic dermatitis OR exp rhinitis) AND exp child) OR ((autoimmun* OR hypersensitiv* OR asthma OR eczema OR atopic dermatitis OR food allerg* OR rhinitis) adj4 (child* OR p?ediatric)).ti,ab | 28321 |
| 5 | (exp animal OR animal experiment.sh OR nonhuman.sh) NOT ((exp animal OR animal experiment.sh OR nonhuman.sh) AND (exp human OR human experiment.sh)) | 408901 |
| 6 | 1 AND 2 | 29 |
| 7 | 6 NOT 5 | 30 |
| 8 | 1 AND 2 AND 3 | 8 |
| 9 | 8 NOT 5 | 8 |
| 10 | 1 AND 2 AND 4 | 4 |
| 11 | 10 NOT 5 | 4 |
| 12 | 1 AND 3 | 26 |
| 13 | 12 NOT 5 | 26 |
| 14 | 7 OR 9 OR 11 OR 13 | 34 |

**PubMed search strategy**

| 1 | (("Antibiotic prophylaxis"[mh] OR "streptococcus agalactiae"[mh]) AND ("labor, obstetric"[mh] OR "parturition"[mh] OR "pregnancy"[mh])) OR "intrapartum antibiotic prophylaxis"[tiab] OR "antibiotic labour"[tiab:~3] OR "antibiotics labour"[tiab:~3] OR "antibiotic labor"[tiab:~3] OR "antibiotics labor"[tiab:~3] OR "antibiotic pregnancy"[tiab:~3] OR "antibiotics pregnancy"[tiab:~3] OR "prenatal antibiotic"[tiab:~3] | [4952](https://pubmed.ncbi.nlm.nih.gov/?term=%22Antibiotic+prophylaxis%22%5Bmh%5D+OR+intrapartum+antibiotic+prophylaxis%2A%5Btiab%5D+OR+%22streptococcus+agalactiae%22%5Bmh%5D+OR+group+b+streptococcus%5Btiab%5D+OR+%22antibiotic%2A+labour%22%5Btiab%3A~2%5D&sort=) |
| --- | --- | --- |
| 2 | ("Gastrointestinal microbiome"[mh] AND ("infant"[mh] OR "child"[mh])) OR  ((Gastrointestinal microbio*[tiab] OR gut microbio*[tiab] OR gut flora[tiab] OR gut microflora[tiab] OR gastrointestinal flora[tiab] OR gastrointestinal microflora[tiab] OR intestinal flora[tiab] OR intestinal microflora[tiab] OR gastric microbio*[tiab] OR enteric bacteria[tiab]) AND (infan*[tiab] OR child*[tiab])) | 9675 |
| 3 | "Pediatric obesity"[mh] OR (("body mass index"[mh] OR "obesity"[mh]) AND "child"[mh]) OR child obesity[tiab] OR childhood obesity[tiab] OR paediatric obesity[tiab] OR pediatric obesity[tiab] | 65253 |
| 4 | (("Autoimmune diseases"[mh] OR "hypersensitivity"[mh] OR "asthma"[mh] OR "eczema"[mh] OR "dermatitis, atopic"[mh] OR "food hypersensitivity"[mh] OR "rhinitis, allergic"[mh]) AND ("child"[mh] OR "infant"[mh])) OR  ((autoimmun*[tiab] OR hypersensitiv[tiab] OR asthma[tiab] OR eczema[tiab] OR atopic dermatitis[tiab] OR food allerg*[tiab] OR rhinitis[tiab]) AND (child*[tiab] OR paediatric*[tiab] OR pediatric*[tiab])) | 189123 |
| 5 | "Animals"[mh] NOT ("animals"[mh] AND "humans"[mh]) | 5355959 |
| 6 | 1 AND 2 NOT 5 | 92 |
| 7 | 1 AND 3 NOT 5 | 34 |
| 8 | 1 AND 4 NOT 5 | 126 |
| 9 | 1 AND 2 AND 3 NOT 5 | 8 |
| 10 | 1 AND 2 AND 4 NOT 5 | 20 |
| 11 | 6 OR 7 OR 8 OR 9 OR 10 | 223 |

**Scopus search strategy reporting**

| 1 | "Intrapartum antibiotic prophylaxis" OR (("group b streptococcus" OR antibiotic*) AND (labo?r OR pregnancy OR prenatal)) | 30007 |
| --- | --- | --- |
| 2 | (("Gastrointestinal microbiome" OR "gut microbiome" OR "gut flora" OR "gut microflora" OR "gastrointestinal flora" OR "gastrointestinal microflora" OR "intestinal flora" OR "intestinal microflora" OR "gastric microbiome" OR "enteric bacteria") AND (infant OR child*)) | 9483 |
| 3 | (("body mass index" OR obesity) AND (child* OR p?ediatric)) | 139167 |
| 4 | (("autoimmune disease" OR autoimmun* OR hypersensitiv* OR "food allerg" OR eczema OR asthma OR "atopic dermatitis" OR rhinitis) AND (child* OR p?ediatric)) | 176355 |
| 5 | 1 AND 2 | 405 |
| 6 | 1 AND 3 | 305 |
| 7 | 1 AND 2 AND 3 | 58 |
| 8 | 1 AND 4 | 701 |
| 9 | 1 AND 2 AND 4 | 74 |
| 10 | 5 OR 6 OR 7 OR 8 OR 9 | 1221 |

**Web of science search strategy**

| 1 | "Intrapartum antibiotic prophylaxis" OR (("group b streptococcus" OR antibiotic*) AND (labo?r OR pregnancy OR prenatal)) | 8508 |
| --- | --- | --- |
| 2 | (("Gastrointestinal microbio"* OR "gut microbio"* OR "gut flora" OR "gut microflora" OR "gastrointestinal flora" OR "gastrointestinal microflora" OR "intestinal flora" OR "intestinal microflora" OR "gastric microbio"* OR "enteric bacteria") AND (infan* OR child*)) | 2744 |
| 3 | ((body mass index OR obesity) AND (child* OR p?ediatric)) | 150925 |
| 4 | (("autoimmune disease" OR autoimmun* OR hypersensitiv* OR "food allerg" OR eczema OR asthma OR "atopic dermatitis" OR rhinitis) AND (child* OR p?ediatric)) | 151319 |
| 5 | 1 AND 2 | 28 |
| 6 | 1 AND 3 | 199 |
| 7 | 1 AND 4 | 363 |
| 8 | 1 AND 2 AND 3 | 2 |
| 9 | 1 AND 2 AND 4 | 8 |
| 10 | 5 OR 6 OR 7 OR 8 OR 9 | 600 |
