# Supplementary material for: Intrapartum Antibiotic Prophylaxis and Child Health Outcomes: A Systematic Review and Meta‐Analysis of Observational Studies
Source: BJOG. 2025 Sep 26;133(4):556–67. doi: 10.1111/1471-0528.70015 (PMC12884238; doi:10.1111/1471-0528.70015)
Supplement: Supplementary file 10 — Table S2: Basic characteristics of eligible studies for infants' gut microbiome outcomes. [file BJO-133-556-s002.docx]

| **Table supplementary 2.** Basic characteristics of eligible studies for infants’ gut microbiome outcomes | | | | | | |
| --- | --- | --- | --- | --- | --- | --- |
| First author (year)  Country | Design  Infant age at follow-up (days/weeks/months) | Mothers exposed to IAP/non-exposed | Tye of IAP | Outcome assessment method | Adjusted variables | Infant gut microbiome outcomes  Mean change (95% CI) |
| Chen et al. (2023)  Canada | Prospective cohort  3 m | 223/541 | Penicillin G | Illumina MiSeq sequencing of V4 regional 16S ribosomal RNA (rRNA) | Breastfeeding, neonatal antibiotic exposure | ● Gut microbiome composition  (figure 5)  ● Gut microbiome Biodiversity  - 0.06 (- 0.22, 0.09) |
| Santos et al. (2023)  Canada | Prospective cohort  10d | 59/184 | Penicillin G | Illumina MiSeq sequencing of V4 regional 16S ribosomal RNA (rRNA) | None | ● Gut microbiome composition  (figure 5)  ● Gut microbiome Biodiversity  - 0.09 (- 0.20, 0.02) |
| Matharu et al. (2022)  Finland | Prospective cohort  3w- 1 y | 24/41 | Penicillin-G, cefuroxime, clindamycin | Illumina MiSeq sequencing of V4 regional 16S ribosomal RNA (rRNA) | None | ● Gut microbiome composition  (figure 5)  ● No changes in gut microbiome Biodiversity |
| Ainonen et a. (2022)  Finland | Prospective cohort  1 y | 27/27 | Penicillin G,  Cefuroxime,  Clindamycin,  Penicillin G+ Cefuroxime | Next-generation sequencing of bacterial 16S ribosomal RNA (rRNA) | None | ● Gut microbiome composition  (figure 5)  ● Gut microbiome Biodiversity  0.0 (- 0.53, 0.53) |
| Coker et al. (2020)  US | Prospective cohort  6 w- 1 y | 29/123 | Penicillin,G, cephalosporins, mixed class | Illumina MiSeq sequencing of V4 regional 16S ribosomal RNA (rRNA) | Breastfeeding, neonatal antibiotic exposure, other prenatal antibiotic exposure | ● Gut microbiome composition  (figure 5)  ● Gut microbiome Biodiversity  - 0.09 (- 0.5, 0.31) |
| Wong et al. (2020)  US | Prospective cohort  2 d | 19/43 | Penicillin G | Illumina MiSeq sequencing of V4 regional 16S ribosomal RNA (rRNA) | Other prenatal antibiotic exposure | Gut microbiome biodiversity was higher in the IAP-exposed compared with control group |
| Nogacka et al. (2017)  Spain | Prospective cohort  2- 90 d | 18/22 | Penicillin G | Illumina MiSeq sequencing of V4 regional 16S ribosomal RNA (rRNA) | None | ● Gut microbiome composition  (figure 5)  ● Gut microbiome biodiversity was reduced in IAP-exposed comparison with control group |
| Stearns et al. (2017)  Canada | Prospective cohort  3d – 3 m | 14/53 | Penicillin G | Illumina MiSeq sequencing of V4 regional 16S ribosomal RNA (rRNA) | None | ● Gut microbiome composition  (figure 5)  ● Gut microbiome Biodiversity  - 0.30 (- 0.89, 0.29) |
| Mazzola et al. (2016)  Italy | Prospective cohort  30 d | 6/7 | Ampicillin | Illumina MiSeq sequencing of V4 regional 16S ribosomal RNA (rRNA) | None | ● Gut microbiome composition  (figure 5)  ● Gut microbiome Biodiversity  - 0.96 (- 2.14, 0.21) |
| Arboleya et al. (2015)  Spain | Prospective cohort  3 m | 3/10 | Ampicillin | Illumina MiSeq sequencing of V4 regional 16S ribosomal RNA (rRNA) | None | Gut microbiome composition  (figure 5) |
| Jaure´guy et al. (2004)  France | Prospective cohort  3 d | 23/23 | Amoxicillin | NR | None | Gut microbiome composition  (figure 5) |
