# Supplementary material for: Intrapartum Antibiotic Prophylaxis and Child Health Outcomes: A Systematic Review and Meta‐Analysis of Observational Studies
Source: BJOG. 2025 Sep 26;133(4):556–67. doi: 10.1111/1471-0528.70015 (PMC12884238; doi:10.1111/1471-0528.70015)
Supplement: Supplementary file 11 — Table S3: Newcastle‐Ottawa quality assessment scale for included studies. [file BJO-133-556-s006.docx]

| **Table supplementary 3.** Newcastle-Ottawa quality assessment scale for included studies | | | | | | | | | |
| --- | --- | --- | --- | --- | --- | --- | --- | --- | --- |
|  | **Selection** | | | | **Comparability** | **Outcome** | | |  |
| **Items** | Representative of the exposed cohort (Iron supplementation among community*) | Selection of non-exposed (same community as exposed*) | Ascertainment of exposure (medical record or self-report*) | Outcome not present at start of study (Early GDM presented or not*) | Comparability of study based on design or analysis (main factor: age* + additional*) | Outcome assessment (blind assessment*/ record linkage*) | Appropriate time for measurement of outcome (24-28 weeks*) | Adequacy of follow-up (all subjects or <10% loss*) | Total score |
| **Child auto-immune disease** | | | |  | | | | | |
| Hutton et al. (2023) | ***** | ***** | ***** | ***** |  | ***** | ***** | ***** | 7 |
| Dhudasia et al. (2021) | * | * | * | * | ** | * | * | * | 9 |
| Hong et al. (2022) | * | * | * | * | ** | * | * | * | 9 |
| Wohl et al. (2015) | * | * | * | * |  | * | * | * | 7 |
| Zhang et al. (2023) | * | * | * | * | ** | * | * | * | 9 |
| Ainonen et al. (2024) | * | * | * | * | ** | * | * | * | 9 |
| **Child obesity** | | | | | | | | | |
| Metz et al. (2020) | * | * | * | * |  | * | * | * | 7 |
| Klancic et al. (2022) | * | * | * | * |  | * | * | * | 7 |
| Hutton et al. (2023) | * | * | * | * |  | * | * | * | 7 |
| Sidell et al. (2023) | * | * | * | * | * | * | * | * | 8 |
| **Gut microbiome** | | | | | | | | | |
| Chen et al. (2023) | * | * | * | * |  | * | * | * | 7 |
| Arboleya et al. (2015) | * | * | * | * |  | * | * | * | 7 |
| Ainonen et a. (2022) |  |  | * | * |  | * | * | * | 5 |
| Nogacka et al. (2017) | * | * | * | * |  | * | * | * | 7 |
| Mazzola et al. (2016) | * | * | * | * |  | * | * | * | 7 |
| Jaure´guy et al. (2004) | * | * | * | * |  | * | * | * | 7 |
| Stearns et al. (2017) | * | * | * | * |  | * | * | * | 7 |
| Coker et al. (2020) | * | * | * | * | ** | * | * | * | 9 |
| Matharu et al. (2021) | * | * | * | * |  | * | * | * | 7 |
| Wong et al. (2020) | * | * | * | * |  | * | * | * | 7 |
| Santos et al. (2023) | * | * | * | * |  | * | * | * | 7 |
