# Supplementary material for: Intrapartum Antibiotic Prophylaxis and Child Health Outcomes: A Systematic Review and Meta‐Analysis of Observational Studies
Source: BJOG. 2025 Sep 26;133(4):556–67. doi: 10.1111/1471-0528.70015 (PMC12884238; doi:10.1111/1471-0528.70015)
Supplement: Supplementary file 12 — Table S4: GRADE profile of IAP and child autoimmune‐related disease, child BMI and BMI z‐score and infant gut microbiome. [file BJO-133-556-s004.docx]

**Table supplementary 4.** GRADE profile of IAP and child autoimmune-related disease, child BMI and BMI z-score and infant gut microbiome

| **Certainty assessment** | | | | | | | **No of participants** | | **Effect** | | **Certainty** | **Importance** |
| --- | --- | --- | --- | --- | --- | --- | --- | --- | --- | --- | --- | --- |
| **№ of studies** | **Study design** | **Risk of bias** | **Inconsistency** | **Indirectness** | **Imprecision** | **Publication bias** | **IAP-Exposed** | **Control** | **Relative Risk** | **(95% CI)** |  |  |
| Child autoimmune-related disease | | | | | | | | | | | | |
| 6 | Non-randomized studies | Not serious ^a^ | Very serious ^b^ | Serious ^c^ | Not serious | None | 12225 | 44969 | RR:1.73 | 1.08 - 2.78 | ⨁◯◯◯  very low | Important |
| Child BMI z-score | | | | | | | | | | | | |
| 3 | Non-randomized studies | Not serious | Very serious | Not serious | Serious ^d^ | None | 1258 | 4918 | SMD: 0.13 | - 0.03 - 0.29 | ⨁◯◯◯  Very low | Important |
| Child BMI | | | | | | | | | | | | |
| 2 | Non-randomized studies | Not serious | Not serious | Not serious | Not serious | None | 22568 | 95416 | SMD: 0.05 | 0.03 - 0.06 | ⨁⨁◯◯  Low | Important |
| Infant gut microbiome biodiversity | | | | | | | | | | | | |
| 7 | Non-randomized studies | Not serious | Not serious | Not serious | Serious | None | 358 | 935 | SMD:  -0.09 | - 0.2 - 0.02 | ⨁◯◯◯  Very low | Important |
|  | | | | | | | | | | | | |

1. More than 20% of studies had low quality and more than 80% of studies didn't have an appropriate score for the main domain.
2. I-squared was more than 50% and the risk of bias domain was very serious.
3. Indirectness of outcome: included studies reported specific allergic outcomes while the outcome of interest in the current review was autoimmune-related diseases
4. The confidence interval is both broad (wide) and crosses the line of no effect, contributing to imprecision.
